# Dimerization of MORC2 through its C-terminal coiled-coil domain enhances chromatin dynamics and promotes DNA repair

**DOI:** 10.1186/s12964-019-0477-5

**Published:** 2019-12-03

**Authors:** Hong-Yan Xie, Tai-Mei Zhang, Shu-Yuan Hu, Zhi-Ming Shao, Da-Qiang Li

**Affiliations:** 1 Shanghai Cancer Center and Institutes of Biomedical Sciences, Shanghai Medical College, Fudan University, Shanghai, 200032 China; 2 Cancer Institute, Shanghai Medical College, Fudan University, Shanghai, 200032 China; 3 Department of Oncology, Shanghai Medical College, Fudan University, Shanghai, 200032 China; 4 Department of Breast Surgery, Shanghai Medical College, Fudan University, Shanghai, 200032 China; 5 Key Laboratory of Breast Cancer in Shanghai, Shanghai Medical College, Fudan University, Shanghai, 200032 China; 6 Key Laboratory of Medical Epigenetics and Metabolism, Shanghai Medical College, Fudan University, Shanghai, 200032 China

**Keywords:** DNA damage response, Chromatin remodeling, MORC2, Coiled-coil domain, Dimerization

## Abstract

Decondesation of the highly compacted chromatin architecture is essential for efficient DNA repair, but how this is achieved remains largely unknown. Here, we report that microrchidia family CW-type zinc finger protein 2 (MORC2), a newly identified ATPase-dependent chromatin remodeling enzyme, is required for nucleosome destabilization after DNA damage through loosening the histone-DNA interaction. Depletion of MORC2 attenuates phosphorylated histone H2AX (γH2AX) focal formation, compromises the recruitment of DNA repair proteins, BRCA1, 53BP1, and Rad51, to sites of DNA damage, and consequently reduces cell survival following treatment with DNA-damaging chemotherapeutic drug camptothecin (CPT). Furthermore, we demonstrate that MORC2 can form a homodimer through its C-terminal coiled-coil (CC) domain, a process that is enhanced in response to CPT-induced DNA damage. Deletion of the C-terminal CC domain in MORC2 disrupts its homodimer formation and impairs its ability to destabilize histone-DNA interaction after DNA damage. Consistently, expression of dimerization-defective MORC2 mutant results in impaired the recruitment of DNA repair proteins to damaged chromatin and decreased cell survival after CPT treatment. Together, these findings uncover a new mechanism for MORC2 in modulating chromatin dynamics and DDR signaling through its c-terminal dimerization.

## Background

DNA is continuously challenged by both endogenous and exogenous genotoxic agents that induce a variety of DNA lesions. Inefficient or inaccurate repair of these genotoxic lesions is intimately implicated in genomic instability and carcinogenesis [1]. To maintain genomic integrity, cells have developed a complex molecular network, termed the DNA damage response (DDR), to detect and repair damaged DNA in the context of chromatin [2]. Naturally, chromatin is a highly condensed structure that presents a significant barrier to the ability of the DNA repair machinery to access and repair DNA lesions [3–5]. Consequently, damaged chromatin must become more accessible to enable DNA repair [5, 6]. Recent work highlights that ATP-dependent chromatin remodeling enzymes and histone modifying enzymes contribute to dynamic changes of chromatin in response to genotoxic stress [7, 8]. However, the underlying mechanisms remain largely unknown.

Microrchidia family CW-type zinc finger protein 2 (MORC2) is a member of the evolutionarily conserved nuclear protein superfamily, which contains an N-terminal catalytically active ATPase module, a central CW-type zinc finger (CW-ZF) domain, a C-terminal chromo-like domain, and four distinct coiled-coil (CC) domains [9, 10]. The ATPase module is composed of gyrase, Hsp90, histidine kinase, and MutL (GHKL) and S5-fold domains, which has been mechanistically linked to gene transcription and DNA repair by remodeling chromatin [11–13]. The CW-ZF domain is present in several chromatin-remodeling proteins [14] and acts as a histone methylation reader [15, 16]. In addition, the C-terminal chromo-like domain is commonly found in eukaryotic chromatin proteins and can recognize methylated peptides in histones and nonhistone proteins [17–19]. These structural features indicate that MORC2 is potentially implicated in chromatin-based processes. Indeed, emerging evidence shows that MORC2 regulates heterochromatin formation and epigenetic gene silencing through an association with human silencing hub (HUSH) complex [20, 21]. In addition, we recently demonstrated that MORC2 facilitates ATPase-dependent chromatin remodeling and efficient DNA repair [13]. However, the detailed mechanism by which MORC2 regulates chromatin dynamics during DDR remains incompletely understood.

In this study, we report that MORC2 loosens histone-DNA interaction and promotes the accessibility of DNA repair machinery to DNA damage lesions, thus facilitating cell survival after DNA damage induced by DNA-damaging chemotherapeutic agent camptothecin (CPT). Moreover, the C-terminal coiled-coil domain of MORC2 is required for its dimerization and the noted biological functions in DDR. These findings provide a novel molecular link between MORC2 and chromatin dynamics in response to DNA damage. Given its emerging roles in tumor progression [22–25] and in resistance to DNA-damaging radio- and chemotherapies [13, 25], MORC2 could be a good candidate target for cancer treatment.

## Results

### MORC2 weakens histone-DNA interaction in cells both under unstressed condition and after DNA damage

**Fig. 1:**
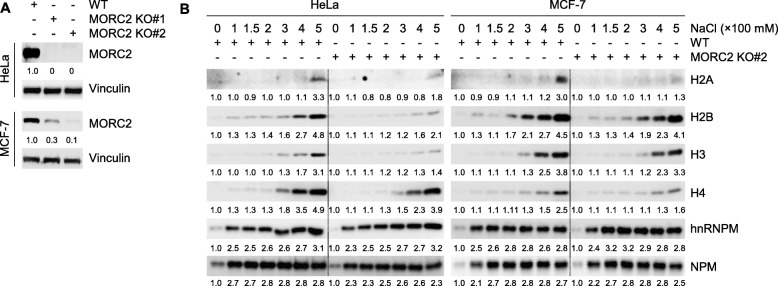
MORC2 attenuates histone and DNA interaction. **a** Knockout of MORC2 in HeLa and MCF-7 cells were carried out by the CRISPR/Cas9 system and validated by immunoblotting analysis with the indicated antibodies. **b** Nuclei were isolated from WT and MOCR2 KO HeLa or MCF-7 cells and subjected to salt solubilization assays. The core histones in salt soluble fractions were detected by immunoblotting using the indicated antibodies. NPM and hnRNPM are shown as loading controls

The evolutionarily conserved ATP-dependent chromatin remodeling enzyme MORC2 has emerging roles in gene transcription and DNA repair [13, 20, 21]. However, how MORC2 modifies chromatin structure remains elusive. To address this question, we knocked out MORC2 using the CRISPR/Cas9 system in HeLa and MCF-7 cells, which are widely used in DNA damage-related studies [26] (Fig. 1a), and then analyzed the effects of MORC2 depletion on the interactions between histone and DNA using salt solubilization assays [27, 28]. To do this, isolated nuclei from wild-type (WT) and MORC2 knockout (KO) cells were fractionated with increasing concentrations of sodium chloride (NaCl), and the core histones in each fraction were detected by immunoblotting. Results showed the core histones (H2A, H2B, H3, and H4) can be extracted from chromatin at lower concentrations of NaCl in WT HeLa and MCF-7 cells as compared with their MORC2 KO counterparts (Fig. 1b). In contrast, knockout of MORC2 did not affect the association of chromatin-related proteins heterogeneous nuclear ribonucleoprotein M (hnRNPM) and nucleophosmin (NPM) [29, 30] with chromatin in the presence of increasing concentrations of NaCl. These results suggest that MORC2 may destabilize nucleosome stability.

**Fig. 2:**
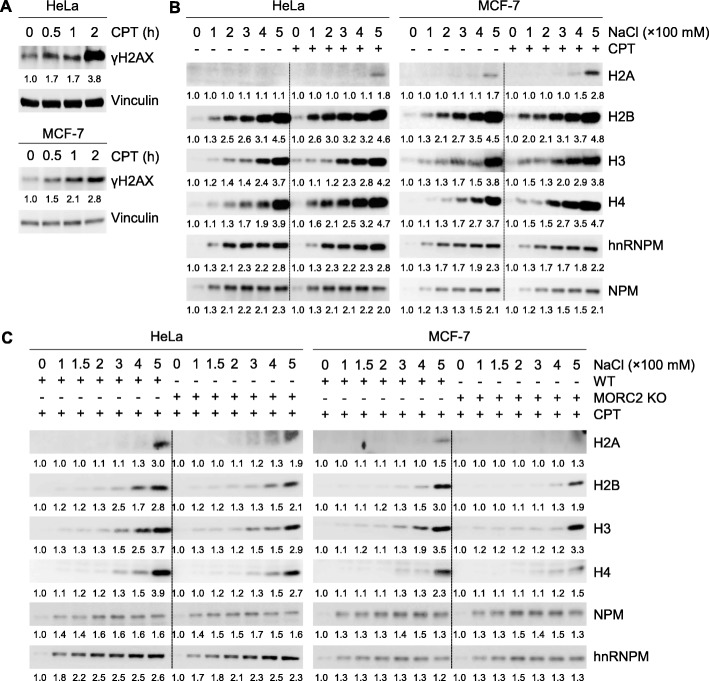
MORC2 is required for altered nucleosome stability after DNA damage. **a** HeLa and MCF-7 cells were treated with or without 8 μM CPT for the indicated times and analyzed by immunoblotting with the indicated antibodies. **b** HeLa and MCF-7 cells were treated with or without 8 μM CPT for 1 h. Nuclei were isolated and subjected to salt solubilization assay as described in Fig. 1b and c. NPM and hnRNPM are shown as loading controls. **c** WT and MORC2 KO HeLa or MCF-7 cells were treated with 8 μM CPT for 1 h. The isolated nuclei were subjected to salt solubilization assay as described above. NPM and hnRNPM are shown as loading controls

Since DNA damage reduces the interaction between DNA and histone [27], we next examined whether MORC2 affects the release of histones from chromatin after DNA damage. For this purpose, we used camptothecin (CPT), an effective DNA-damaging chemotherapeutic drug by specifically targeting DNA topoisomerase I [31], to induce DNA damage. As shown in Fig. 2a, treatment of HeLa and MCF-7 cells with CPT can effectively induce DNA damage in a time-dependent manner, as evidenced by enhanced expression of phosphorylated H2AX (termed γH2AX), a sensitive marker for DNA damage [32]. Then, we isolated cell nuclei from HeLa and MCF-7 cells with or without CPT treatment and performed salt solubilization assays as described above. Results showed the core histones can be isolated at lower salt concentrations in CPT-treated HeLa and MCF-7 cells compared to DMSO treated cells (Fig. 2b), supporting the notion that the stability of the histone-DNA interaction is reduced by DNA damage [27]. More importantly, knockout of MORC2 attenuated DNA damage-induced disassociation of core histones from chromatin (Fig. 2c). These data suggests that MORC2 is required for altered nucleosome stability after DNA damage.

### MORC2 promotes the recruitment of DNA repair proteins to damaged chromatin and decreases cellular sensitivity to DNA-damaging chemotherapeutic agents

**Fig. 3:**
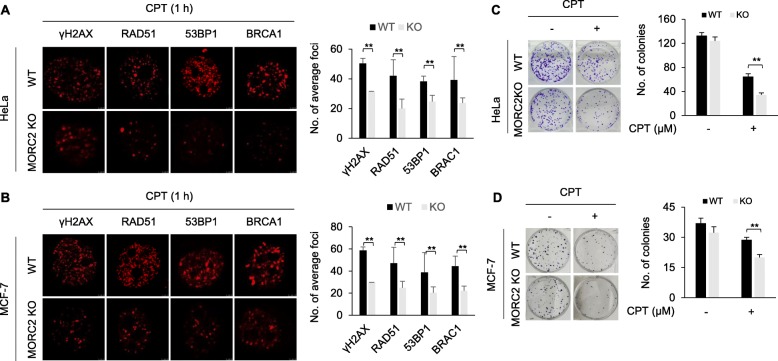
MORC2 promotes the recruitment of DNA repair proteins to DNA damage sites and cell survival in response to DNA damage. **a**-**b** WT and MORC2 KO HeLa (**a**) or MCF-7 (**b**) cells were treated with 8 μM CPT for 1 h. Immunofluorescent staining was carried out with the indicated antibodies. Quantitative results were determined by the average foci from 8 representative cells. **c**-**d** WT and MOCR2 KO HeLa (**c**) and MCF-7 (**d**) cells were treated with or without 4 μM CPT and were subjected to clonogenic survival assays. **, *p* < 0.01

According to the “access-repair-restore” model [6], open chromatin structure is required for DNA repair proteins to access DNA damage sites [3, 33]. Given that MORC2 alters chromatin structure during DDR, we next examined whether knockout of MORC2 affects the recruitment of DNA repair proteins to damaged chromatin. Immunofluorescent staining demonstrated that depletion of MORC2 in both HeLa and MCF-7 cells impaired the focal formation of γH2AX and attenuated subsequent recruitment of DNA repair proteins, such as breast cancer type 1 susceptibility protein (BRCA1), p53-binding protein 1 (53BP1), and RAD51, to DNA damage sites (Fig. 3a and b). To investigate the potential effects of MORC2 on DNA repair, WT and MORC2 KO HeLa and MCF-7 cells were treated with or without CPT and their clonogenic survival was examined. Interestingly, we found that knockout of MORC2 decreased cell survival after CPT treatment as compared with its WT cells (Fig. 3c and d). Consistently, knockout of MORC2 also impaired cell survival in HeLa cells after treatment with another commonly used DNA-damaging agent methyl methanesulfonate (MMS) [34] (Additional file 1: Figure S1). To further validate these findings, we next analyzed the relationship between MORC2 expression levels and the prognosis of breast cancer patients who received chemotherapy in publicly available Kaplan-Meier plotter database for breast cancer (http://kmplot.com). Results showed that high levels of MORC2 were associated with poor recurrence-free survival (RFS) and distant metastasis-free survival (DMFS) of breast cancer patients received chemotherapy (Additional file 1: Figure S2). Collectively, these results suggest that MORC2 promotes DNA repair and its expression levels are associated with resistance to DNA-damaging chemotherapeutic agents.

### MORC2 can homodimerize through its C-terminal coiled-coil domain

**Fig. 4:**
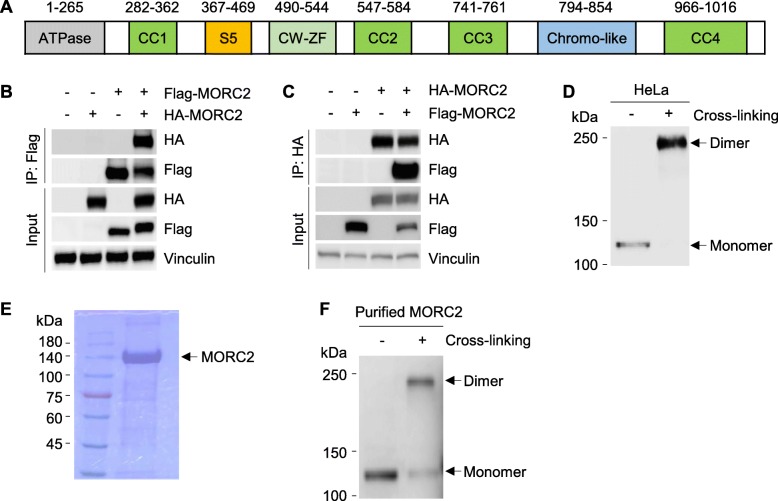
MORC2 can form a homodimer. **a** Schematic diagrams of the domain structure of human MORC2. CC, coiled-coil; CW-ZF, CW-type zinc finger. **b**-**c** HEK293T cells were transfected with Flag-MORC2, HA-MORC2 alone or in combination. After 48 h of transfection, total cellular lysates were subjected to IP analysis with an anti-Flag (**b**) or an anti-HA (**c**) antibody, followed by immunoblotting with the indicated antibodies. **d** HEK293T cells were transfected with HA-MORC2. After 48 h of transfection, total cellular lysates were subjected to cross-linking assay. **e** Purified MORC2 was visualized by coomasie brilliant blue staining. **f** Purified MORC2 was subjected to cross-linking assay and immunoblotting

As mentioned earlier, MORC2 contains four distinct coiled-coil domains (Fig. 4a). Given that many coiled-coil domains function as a dimerization motif [35], we next tested whether MORC2 could form a dimer. To do this, HEK293T cells were transfected with Flag-MORC2, HA-MORC2 alone or in combination. After 48 h of transfection, total cell lysates were subjected to reciprocal IP assays with an anti-Flag or an anti-HA antibody. Immunoblotting analysis showed that Flag-MORC2 interacted with HA-MORC2 when co-expressed (Fig. 4b and c), highlighting the ability of MORC2 to homodimerize in vivo. In support of this notion, cross-linking assays using total cellular lysates of HeLa cells showed that cross-linked MORC2 shifted to the locus that is double of MORC2 molecular weight (Fig. 4d). To exclude the possibility that the shift of cross-linked MORC2 was due to the inference of other proteins that interact with MORC2, we purified Flag-MORC2 protein from HEK293T cells (Fig. 4e) and then performed cross-linking assays. Immunoblotting analysis showed that MORC2 indeed formed a dimer (Fig. 4f). Together, these results demonstrated that MORC2 can form a homodimer.

**Fig. 5:**
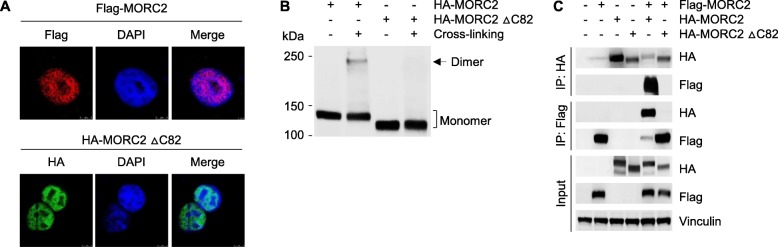
The C-terminal coiled-coil domain of MORC2 is required for its dimer formation. **a** HEK293T cells were transfected with either Flag-MORC2 or HA-MORC2 ΔC82. After 48 h of transfection, immunofluorescent staining was carried out using an anti-Flag or an anti-HA antibody. Nuclei were counterstained with DAPI. **b** HEK293T cells were transfected with HA-MORC2 and HA-MORC2 ∆C82. After 48 h of transfection, total cellular lysates were subjected to cross-linking assay, followed by immunoblotting with an anti-Flag antibody. **c** HEK293T cells were transfected with HA-MORC2, HA-MORC2 ∆C82 alone or in combination with Flag-MORC2. After 48 h of transfection, total cellular lysates were subjected to IP analysis with an anti-Flag or an anti-HA antibody, followed by immunoblotting with the indicated antibodies

The coiled-coil domain is responsible for the oligomerization of proteins in a highly specific manner [36]. Protein sequence analysis predicates that the highly conserved C-terminal 82 amino acids (residues 950–1032) in MORC2 (Additional file 1: Figure S3), in which contains a coiled coil domain (residues 966–1016) (https://www.uniprot.org/uniprot/Q9Y6X9), may be vital for MORC2 dimerization. To test this notion, we generated a deletion truncate in which the C-terminal 82 amino acids were deleted (HA-MORC2 ∆C82). Immunofluorescent staining revealed that deletion of the C-terminal 82 amino acids in MORC2 did not affect its subcellular localization (Fig. 5a). Cross-linking assays showed that HA-MORC2 ∆C82 failed to form a dimer as compared with its WT counterpart (Fig. 5b), indicating that the C-terminal 82 amino acids in MORC2 are required for its dimerization. To further confirm this finding, HEK293T cells were transfected with HA-MORC2, HA-MORC2 ∆C82 alone or in combination with Flag-MORC2. After 48 h of transfection, total cellular lysates were subjected to IP analysis with an anti-Flag or an anti-HA antibody. Immunoblotting analysis showed that HA-MORC2, but not HA-MORC2 ∆C82, can interact with Flag-MORC2, and *vice eras* (Fig. 5c). These data suggests that MORC2 can form a dimer and that the C-terminal coiled-coil domain is critical for MORC2 dimerization.

### DNA damage enhances MORC2 dimerization

**Fig. 6:**
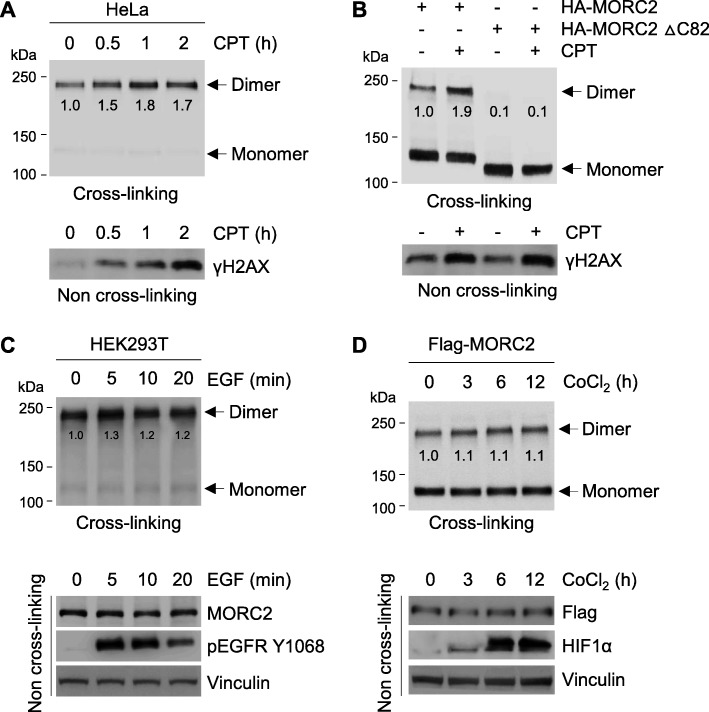
MORC2 dimerization is enhanced in response to DNA damage. **a** HeLa cells were treated with 8 μM CPT for the indicated times. Lysates were subjected to cross-linking assays, followed by immunoblotting analysis with the indicated antibodies (upper panel). The expression levels of γH2AX in lysates without chemical cross-linking are shown as a control for CPT-induced DNA damage (bottom panel). **b** HEK293T cells were transfected with HA-MORC2 and HA-MORC2 ∆C82. After 48 h of transfection, total cellular lysates were subjected to cross-linking assay. Immunoblotting analysis was carried out with the indicated antibodies (upper panel). The expression levels of γH2AX in lysates without chemical cross-linking are shown as a control for CPT-induced DNA damage (bottom panel). **c** HeLa cells were treated with 20 ng/mL EGF for the indicated times and subjected to cross-linking assay (upper panel). The expression levels of phosphorylated EGFR (Y1068) in lysates without chemical cross-linking are shown as a control for the activation of downstream signaling by EGF (bottom panel). **d** HeLa cells were treated with 200 μM CoCl_2_ for the indicated times and subjected to cross-linking assay (upper panel). The expression levels of hypoxia-inducible factor 1α (HIF1α) in lysates without chemical cross-linking are shown as a control for the activation of hypoxia signaling by CoCl_2_ (bottom panel)

To investigate whether DNA damage could affect MORC2 dimerization, we treated HeLa cells with CPT for the indicated times. Then, total cellular lysates were subjected to cross-linking assays and analyzed by immunoblotting with the indicated antibodies. Results showed that MORC2 dimerization was enhanced in cells treated with CPT (Fig. 6a). Consistently, CPT treatment also enhanced the dimer formation of exogenously expressed HA-MORC2, but not HA-MORC2 ∆C82 (Fig. 6b). Given that other extracellular signals, such as epidermal growth factor (EGF) [37] and hypoxia [38], can induce protein dimer formation, we next investigated the effects of EGF and hypoxia mimetic cobalt chloride (CoCl_2_) [39] on MORC2 dimerization. Results showed that treatment of HeLa cells with either EGF or CoCl_2_ did not significantly affect MORC2 dimerization (Fig. 6c and d, respectively). These results collectively suggest that MORC2 dimerization is enhanced in response to DNA damage.

### MORC2 dimerization is required for altered nucleosome stability after DNA damage and subsequent DNA repair signaling

**Fig. 7:**
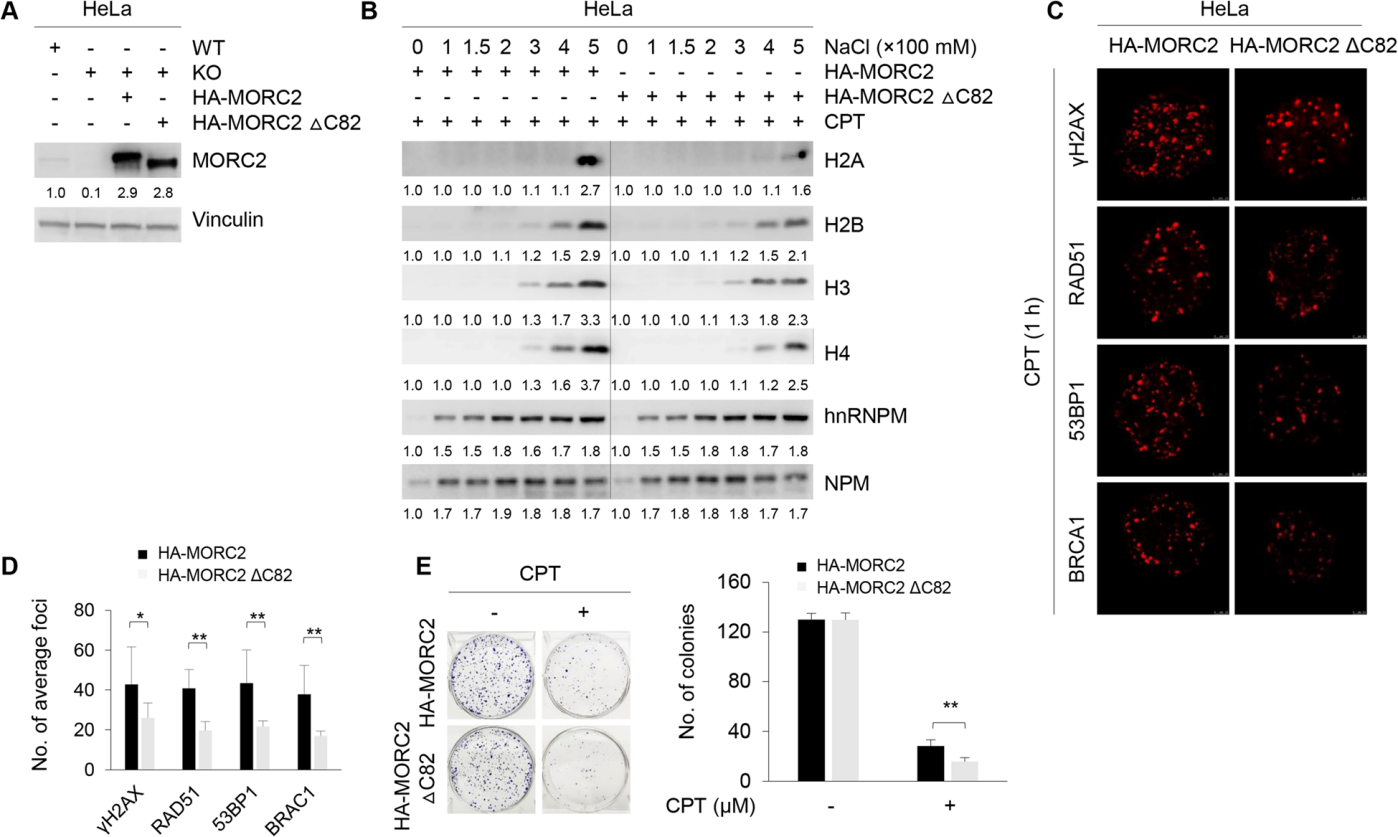
MORC2 dimerization is implicated in altered nucleosome stability after DNA damage and subsequent DDR signaling. **a** HA-MORC2 and HA-MORC2 ∆C82 in pCDH-CMV-MCS-EF1-copGFP expression vector were re-expressed in MORC2 KO HeLa cells by lentiviral infection. After 48 h of infection, GFP-positive cells were selected by FACS. Expression levels of MORC2 in established stable cell lines were verified by immunoblotting analysis. **b** HeLa cells stably expressing HA-MORC2 and HA-MORC2 ∆C82 were treated with 8 μM CPT for 1 h and subjected to salt solubilization assays as described above. Immunoblotting analysis was carried out with the indicated antibodies. NPM and hnRNPM were used as loading controls. **c**-**d** HeLa cells stably expressing HA-MORC2 and HA-MORC2 ∆C82 were treated with 8 μM CPT for 1 h. Immunofluorescent staining was carried out using the indicated antibodies (**c**). Quantitative results (foci per cell) are shown in **d**. Eight representative cells stained with the individual antibody were counted. **e** HeLa cells stably expressing HA-MORC2 and HA-MORC2 ∆C82 were treated with CPT at the indicated doses and subjected to clonogenic survival assays. **, *p* < 0.01

To determine the role of MORC2 dimerization in altered nucleosome stability after DNA damage, we first stably expressed HA-MORC2 and HA-MORC2 ∆C82 in MORC2 KO HeLa cells by lentiviral infection and validated the expression status of exogenously expressed MORC2 by immunoblotting (Fig. 7a). Salt solubilization assays showed that the core histones were extracted from chromatin at lower concentrations of NaCl in HA-MORC2 expressing cells as compared with HA-MORC2 ∆C82 expressing cells (Fig. 7b). In support of this notion, immunofluorescent staining demonstrated that expression of HA-MORC2 ∆C82 impaired the recruitment of DNA repair proteins, such as BRCA1, 53BP1, and Rad51, to sites of DNA damage after CPT treatment (Fig. 7c and d). Moreover, cells expressing HA-MORC2 ∆C82 had decreased cell survival after CPT treatment compared to HA-MORC2 expressing cells (Fig. 7e). These data indicates that dimerization of MORC2 is required for the noted functions of MORC2 in DDR signaling, including chromatin decompaction and the accumulation of DNA repair proteins at sites of DNA damage.

## Discussion

**Fig. 8:**
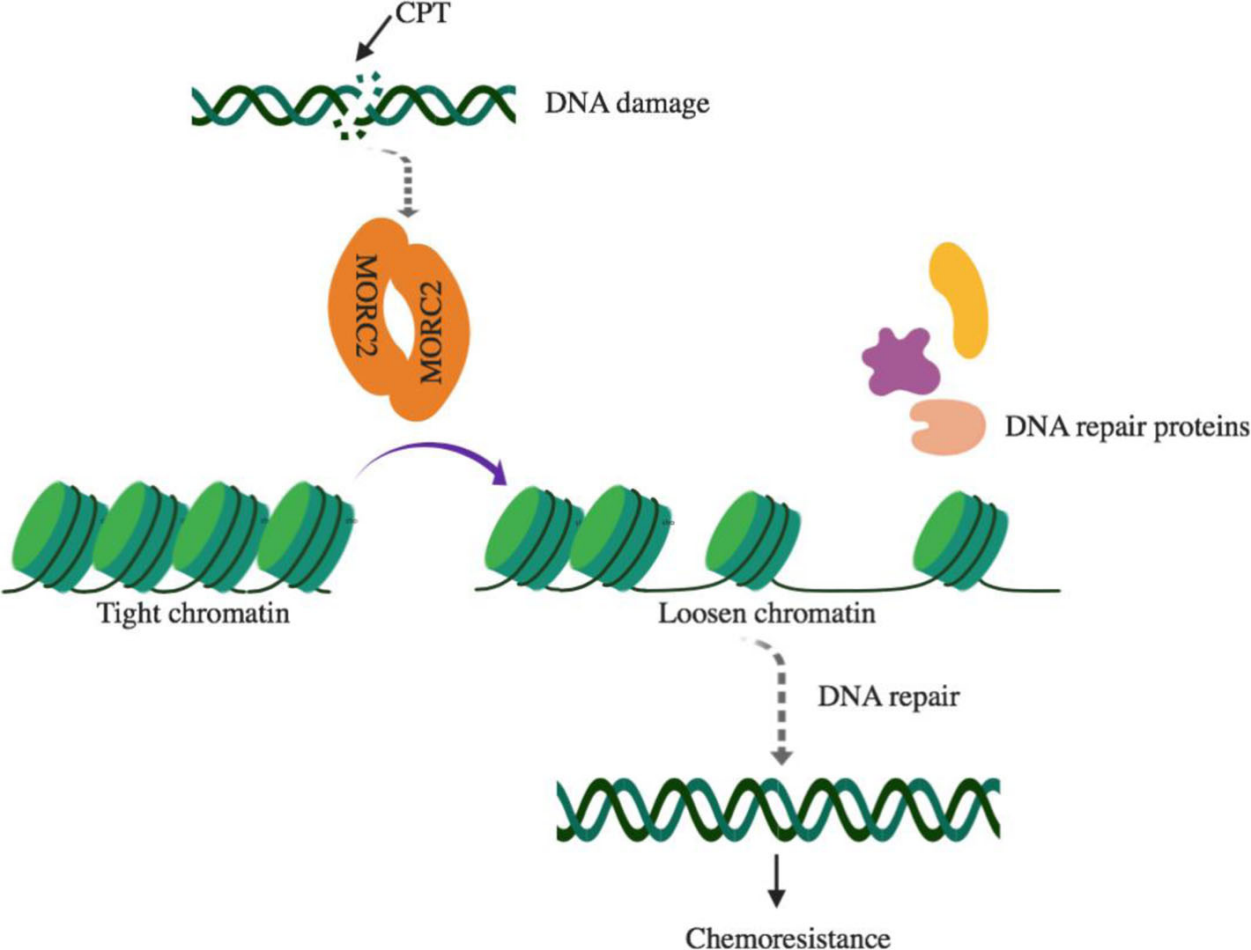
The proposed working model

In this study, we provide biochemical and functional evidence for the mechanistic link between MORC2 dimerization and the alteration of chromatin structure during DDR (Fig. 8). Alterations in chromatin structure are closely linked to chromatin-based biological processes such as gene transcription and DDR. MORC2 as an emerging chromatin remodeling protein has been shown to regulate epigenetic gene silencing [20, 21] and DDR [13]. As histone-DNA interactions within the nucleosomes can be transiently broken by ATP-dependent nucleosome remodelers [40], we analyzed the ability of histones to disassociate from chromatin in MORC2-depleted cells treated with or without DNA-damaging chemotherapeutic drug CPT by salt solubilization assays. We found that MORC2 attenuates the histone-DNA interaction, and this effect was further enhanced by CPT-induced DNA damage (Figs. 1 and 2). These findings are support of the notion that dynamic remodeling of chromatin structure is essential for efficient DNA repair [5, 6]. In contrast to the functional role of MORC2 in relaxing chromatin during DDR, MORC2 has recently been documented to be recruited to heterochromatic loci by HUSH complex to compact chromatin, thus leading to suppression of gene transcription [20]. Given that proteins may have distinct functions in term of its diverse loci on chromatin, MORC2 could compact or decompact chromatin under specific conditions at specific loci by specifically interacting with different complexes, although the underling mechanisms need to be further explored. In support of this notion, several heterochromatin proteins, such as heterochromatin protein 1 (HP1) [41, 42] and Krüppel-associated box (KRAB) domain-associated protein 1 (KAP1) [43, 44], have been shown to act as transcriptional repressors but promote DNA repair.

Coiled-coil domain plays essential roles in protein assembly and molecular recognition. For instance, the coiled-coil domain of human ATR-interacting protein (ATRIP) contributes to self-dimerization in vivo, which is important for cellular response to replication stress and DNA damage [35]. Dimerization of CtIP, a BRCA1- and CtBP-interacting protein, is mediated by an N-terminal coiled-coil motif [45]. As MORC2 protein contains four distinct coiled-coil domains, we proposed that MORC2 may form a dimer. In support of this notion, co-IP and glutaraldehyde cross-linking assays demonstrated that MORC2 indeed forms a dimer (Fig. 4) and that the C-terminal coiled-coil domain is essential for MORC2 dimerization (Fig. 5). Unfortunately, we failed to map the specific sites responsible for MORC2 dimerization although we worked hard on this point (data not shown). Interestingly, we found for the first time that MORC2 dimerization is enhanced in response to DNA damage (Fig. 6). In contrast, growth factor EGF and CoCl_2_, a mimic of hypoxia, did not significantly affect dimer formation of MORC2. In addition, functional rescue experiments revealed that MORC2 dimerization is required for MORC2-mediated destabilization of histone and DNA interaction and subsequent DDR signaling (Fig. 7). Nevertheless, the functional and mechanistic roles for MORC2 dimerization in DDR remain to be explored.

In summary, findings presented here suggest that MORC2 can form a dimer through its C-terminal coiled-coil domain, which is regulated by DNA damage signaling and is implicated in DNA damage repair (Fig. 8). Given that MORC2 is upregulated in multiple types of human cancer [46] and contributes to cancer growth and progression [22–25] as well as cellular response to ionizing radiation [13] and chemotherapeutic drugs [25], these findings broaden our understanding of the functional roles of MORC2 in DDR and highlight MORC2 as a potential target for cancer treatment.

## Methods

### Cell culture and reagents

HeLa, MCF-7, and HEK293T cell lines were obtained from the Bank of Type Culture Collection of Chinese Academy of Sciences (Shanghai, China). All of them were authenticated by short tandem repeat profiling and cultured in high-glucose DMEM media containing 10% fetal bovine serum (Gibco, Carlsbad, USA). All chemicals or regents were purchased from Sigma-Aldrich (St. Louis., USA) unless otherwise noted.

### DNA constructs, transfection, and lentiviral infection

Myc-DDK-MORC2 expression vector was purchased from Origene (Rockville, USA). To generate Flag-tagged full-length MORC2 expression vectors, MORC2 cDNA was amplified by PCR and subcloned into the lentiviral vector pCDH-CMV-MCS-EF1-Puro (System Biosciences, Mountain View, USA). To generate HA-MORC2 and HA-MORC2 ∆C82 (deletion of C-terminal 82 amino acid), MORC2 cDNA was amplified by PCR and subcloned into either pCDH-CMV-MCS-EF1-Puro or pCDH-CMV-MCS-EF1-copGFP lentiviral vectors (System Biosciences). All constructs were verified by DNA sequencing (HuaGene Biotech, Shanghai, China). The primers used for molecular cloning and the expression constructs used in this study are listed in Additional file 1: Tables S1 and S2, respectively.

Transient plasmid transfection was carried out using Neofect DNA transfection reagent (TengyiBio, Shanghai, China) according to the manufacturer’s protocol. Knockout (KO) of MORC2 was performed using the CRISPR/Cas9 system [26] with LentiCas9-Blast and lentiGuide-Puro vectors (Addgene, Cambridge, USA). The short guide RNA (sgRNA) sequences targeting MORC2 are listed in Additional file 1: Tables S3*.* To re-express HA-MORC2 and HA-MORC2 ∆C82 in MORC2 KO cells, individual lentiviral expression vector, along with packaging plasmid mix, was transfected into HEK293T cells. After 48 h of transfection, the viruses in supernatant were collected and used to infect MORC2 KO cells. Sorting of GFP-positive cells was carried out by Fluorescence Activated Cell Sorting (FACS) and immunoblotting was used to further validate the positive cells.

### Antibodies, immunoblotting, immunoprecipitation, and immunofluorescence

The detailed information for primary antibodies is provided in Additional file 1: Table S4*.* For immunoblotting analysis, cells were lysed in the modified RIPA buffer (50 mM Tris-HCl, pH 7.4, 1% NP-40, 0.25% sodium deoxycholate, 1 mM EDTA and 150 mM NaCl) containing 1 × protease inhibitor cocktail and 1 × phosphatase inhibitor cocktail (Bimake, Houston, USA). Proteins were quantified using the bicinchoninic acid assay (Yeasen, Shanghai, China), resolved by SDS-PAGE, and transferred onto PVDF membrane (Millipore, Billerica, USA). Antibody detection was carried out using enhanced chemiluminescence (Yeasen). The immunoblotting data was quantified using Image J software, and the expression levels of proteins were normalized to those of corresponding controls in each panel.

For immunoprecipitation (IP) analysis, cells were lysed in NP-40 lysis buffer (50 mM Tris-HCl, pH 8, 150 mM NaCl, 0.5% NP-40, 10% glycerol, 2 mM MgCl_2_, and 1 mM EDTA), and total 1–2 mg of exogenously expressed proteins were incubated anti-Flag or anti-HA magnetic beads (Bimake) overnight at 4 °C to pull down the protein-antibody complex. The resulting complexes were subjected to immunoblotting analysis.

For immunofluorescent staining, cells were fixed in 4% paraformaldehyde, permeabilized in 0.1% Triton X-100, and blocked in 5% normal goat serum in PBST. Cells were incubated with primary antibodies, washed three times in PBST, and then incubated with the appropriate secondary antibody conjugated with 555-Alexa (red) or 488-Alexa (green) (Cell Signaling Technology, Danvers, USA), respectively. DNA staining was conducted using fluoroshield mounting medium with DAPI (Abcam, Cambridge, USA). Microscopic analyses were performed using a Leica SP5 confocal laser scanning microscopy (Leica Microsystems, Buffalo Grove, USA).

### Salt solubilization assays

Salt solubilization assays were performed as described previously [27, 28]. Briefly, nuclei were isolated from 2 × 10^6^ cells using hypotonic lysis and were incubated in non-denaturing extraction buffers (20 mM Tris-Cl, pH 7.6, 5% glycerol) supplemented with 100 to 500 mM NaCl in the presence of protease inhibitors for 5 min. Cell nuclei were harvested by centrifugation at 700 g for 25 min. Salt soluble fractions were obtained by centrifugation, resolved by SDS-PAGE, and analyzed by immunoblotting using the indicated antibodies.

### Glutaraldehyde cross-linking assays

Cells were lysed with the NP40 buffer and quantified as described above. Then, total cell lysates were cross-linked with 0.05% (w/v) glutaraldehyde (Sigma) on ice for 5 min and terminated by adding 1 M glycine for 15 min at room temperature. After that, the protein samples were analyzed by 8% SDS-PAGE and immunoblotting with the indicated antibodies.

### Protein purification from mammalian cells

Purification of Flag-MORC2 from HEK293T cells was performed as described previously [47]. Briefly, full-length Flag-MORC2 was transfected into HEK293T cells. After 48 h of transfection, cells were lysed in the modified RIPA buffer containing 1 × protease inhibitor cocktail and 1 × phosphatase inhibitor cocktail (Bimake). The clarified supernatant was subjected to pull-down assays using anti-Flag affinity gel (Bimake), and the enriched proteins were eluted using an elution buffer containing 1 mg/ml 3× Flag peptide, 25 mM Tris, pH 8.0, and 100 mM NaCl. The purified protein was used for chemical cross-linking assays.

### Cell viability and clonogenic survival assays

For cell viability assays, cells were seeded in 96-well plates (1000 cells/well) in triplicate and cell viability was analyzed using Cell Counting Kit-8 (CCK-8) kit (Dojindo Laboratories, Kumamoto, Japan). For clonogenic survival assays, cells were seeded in 6-well plates (1000 cells/well) in triplicate and cultured under normal growth conditions for 1–2 weeks. Colonies were stained with 1% crystal violet and counted using an inverted microscope.

### Statistical analysis

All data are presented as the mean ± standard deviation from at least three independent experiments. The Student’s *t*-test was used for assessing the difference between individual groups and *p* ≤ 0.05 was considered statistically significant.

## Supplementary information

**Additional file 1: Figure S1.** Knockout of MORC2 enhanced cellular sensitivity to MMS. **Figure S2.** The expression levels of MORC2 are negatively associated with RFS and DMFS of breast cancer patients who received chemotherapy. **Figure S3.** The C-terminal 82 amino acid sequence of MORC2 is highly conserved among multiple species. **Tables S1.** Primers used for molecular cloning of expression vectors. **Tables S2.** Information of expression vectors used in this study. **Table S3.** sgRNAs targeting for MORC2 used in this study. **Tables S4.** Information for primary antibodies used in this study.

## Data Availability

All data generated or analysed during this study are included in this published article.

## Abbreviations

53BP1: p53-binding protein 1
BRCA1: breast cancer type 1 susceptibility protein
CC: coiled coil
CPT: camptothecin
DDR: DNA damage response
KO: knockout
MORC2: microrchidia family CW-type zinc finger protein 2
WT: wild-type
