# Supplementary material for: Dimerization of MORC2 through its C-terminal coiled-coil domain enhances chromatin dynamics and promotes DNA repair
Source: Cell Commun Signal. 2019 Dec 3;17:160. doi: 10.1186/s12964-019-0477-5 (PMC6892150; doi:10.1186/s12964-019-0477-5)
Supplement: Supplementary file 1 — Additional file 1: Figure S1. Knockout of MORC2 enhanced cellular sensitivity to MMS. Figure S2. The expression levels of MORC2 are negatively associated with RFS and DMFS of breast cancer patients who received chemotherapy. Figure S3. The C-terminal 82 amino acid sequence of MORC2 is highly conserved among multiple species. Tables S1. Primers used for molecular cloning of expression vectors. Tables S2. Information of expression vectors used in this study. Table S3. sgRNAs targeting for MORC2 used in this study. Tables S4. Information for primary antibodies used in this study. [file 12964_2019_477_MOESM1_ESM.pdf]

Supplementary Information for

Xie et al. **Dimerization of MORC2 through its C-terminal coiled-coil domain enhances chromatin dynamics and promotes DNA repair**

The Supplementary Information includes

1. Supplementary Figures S1-S3
2. Supplementary Figure legends
3. Supplementary Tables S1-S4

Supplementary Figure Legends

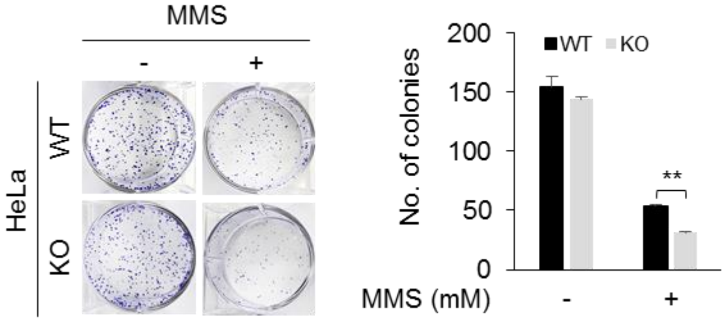

Figure S1. Knockout of MORC2 enhanced cellular sensitivity to MMS

WT and MOCR2 KO HeLa cells were treated with or without 0.2 mM MMS and subjected to clonogenic survival assays. The representative images of survival clones are shown in left panel and the corresponding quantitative results are shown in right panel. **, $p < 0.01$.

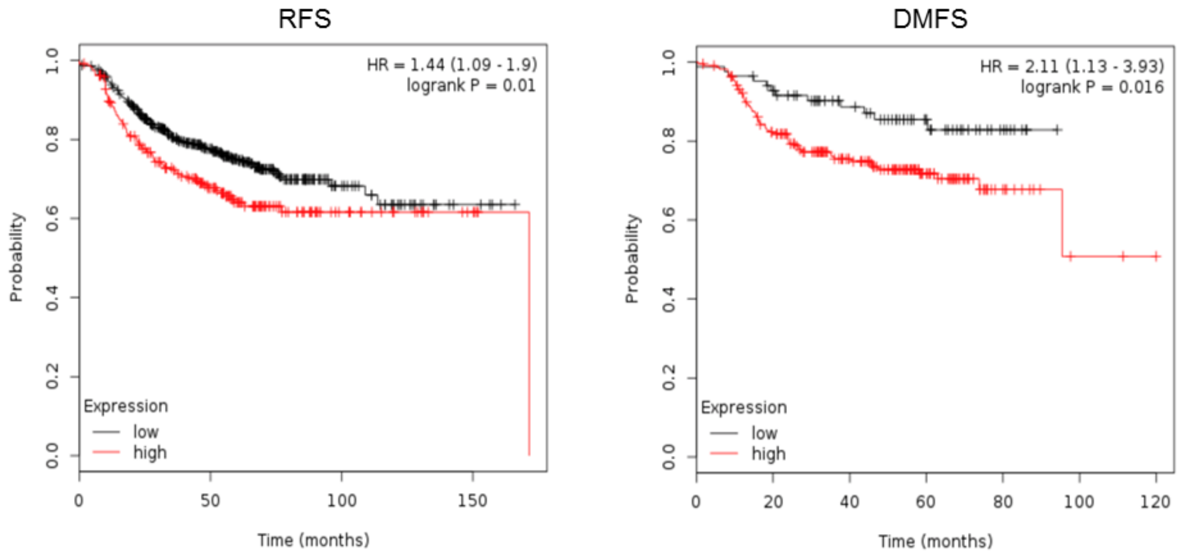

Figure S2. The expression levels of MORC2 are negatively associated with RFS and DMFS of breast cancer patients who received chemotherapy.

Kaplan-Meier curves for recurrence-free survival (RFS) (left) and distant metastasis-free survival (DMFS) (right) of breast cancer patients who received chemotherapy.

```
    Human NP_001290185.1   SAMNSDELISFPLKEYFKQYEVGLQNLCNSYQSRADSRAKASEESLRTSERKLRETEEKL   994
    Sheep XP_011968148.1   SAMNSDELISFPLKEYFKQYEVGLQNLCHSYQSRADSRAKASEESLRTSERKLRETEEKL   999
      Cow XP_015331066.1   SAMNSDELISFPLKEYFKQYEVGLQNLCHSYQSRADSRAKASEESLRTSERKLRETEEKL   1000
Troglodyte XP_016794460.1  SAMNSDELISFPLKEYFKQYEVGLQNLCNSYQSRADSRAKASEESLRTSERKLRETEEKL   994
Orangutan XP_024095432.1   SAMNSDELISFPLKEYFKQYEVGLQNLCNSYQSRADSRAKASEESLRTSERKLRETEEKL   994
   Monkey XP_015005504.1   SAMNSDELISFPLKEYFKQYEVGLQNLCNSYQSRADSRAKASEESLRTSERKLRETEEKL   994
  hamster XP_016824078.1   SAMNSEELISFPLKEYFKQYEVGLQNLCHSYQSRADSRAKASEESLRTSEKKLRETEEKL   890
      Pig XP_005670910.1   SAMNSEELISFPLKEYFKQYEVGLQNLCHSYQSRADSRAKASEESLRTSERKLRETEEKL   995
     Deer XP_020727311.1   SAMNSDELISFPLKEYFRQYEVGLQNLCHSYQSRADSRAKASEESLRTSERKLRETEEKL   1004
     Goat XP_017916461.1   SAMNSDELISFPLKEYFKQYEVGLQNLCHSYQSRADSRAKASEESLRTSERKLRETEEKL   1000
  Buffalo XP_025123123.1   SAMNSDELISFPLKEYFKQYEVGLQNLCHSYQSRADSRAKASEESLRTSERKLRETEEKL   1000
     Fish JAR35272.1       SAMNSDELISFPLKEYFKQYEVGLQNLCNSYQSRADSRAKASEESLRTSERKLRETEEKL   1966
   Cattle XP_019832793.1   SAMNSDELISFPLKEYFKQYEVGLQNLCHSYQSRADSRAKASEESLRTSERKLRETEEKL   1000
  Polecat XP_004766471.1   SAMNSDELISFPLKEYFKQYEVGLQNLCHSYQSRADSRAKASEESLRTSERKLRETEEKL   995
                           *****:***********:**********:**********************:********

    Human NP_001290185.1   QKLRTNIVALLQKVQEDIDINTDDELDAYIEDLITKGD   1032
    Sheep XP_011968148.1   QKLRTNIVALLQ---KDLDINTDDELDAYIEDLITKGD   1034
      Cow XP_015331066.1   QKLRTNIVALLQ---KDLDINTDDELDAYIEDLITKGD   1035
Troglodyte XP_016794460.1  QKLRTNIVALLQKVQEDIDINTDDELDAYIEDLITKGD   1032
Orangutan XP_024095432.1   QKLRTNIVALLQKVQEDIDINTDDELDAYIEDLITKGD   1032
   Monkey XP_015005504.1   QKLRTNIVALLQKVQEDIDINTDDELDAYIEDLITKGD   1032
  hamster XP_016824078.1   QKLRTNIVALLQKVQEDIDINTDDELDAYIEDLITKGD   928
      Pig XP_005670910.1   QKLRTNIVALLQKVQEDIDINTDDELDAYIEDLITKGD   1033
     Deer XP_020727311.1   QKLRTNIVALLQ---KDIDINTDDELDAYIEDLITKGD   1039
     Goat XP_017916461.1   QKLRTNIVALLQ---KDLDINTDDELDAYIEDLITKGD   1035
  Buffalo XP_025123123.1   QKLRTNIVALLQ---KDLDINTDDELDAYIEDLITKGD   1035
     Fish JAR35272.1       QKLRTNIVALLQKVQEDIDINTDDELDAYIEDLITKGD   2004
   Cattle XP_019832793.1   QKLRTNIVALLQ---KDLDINTDDELDAYIEDLITKGD   1035
  Polecat XP_004766471.1   QKLRTNIVALLQKVQEDIDINTDDELDAYIEDLITKGD   1033
                           ************   :*:********************
```

Figure S3. The C-terminal 82 amino acid sequence of MORC2 is highly conserved among multiple species.

Supplementary Tables

Tables S1. Primers used for molecular cloning of expression vectors

Genes	Primers	Sequences
Flag-MORC2	Forward	ACCTCCATAGAAGATCTAGAGCCGCCACCATGATGGCTTTCACAAATTACAGCAGT
	Reverse	GATCCATTTAAATTCGAATTCTTACTTATCGTCGTCATCCTTGTAATCGTCCCCCTTGGTGATGAGGTCCT
HA-MORC2	Forward	ACCTCCATAGAAGATCTAGAGCCGCCACCATGATGGCTTTCACAAATTACAGCAGT
	Reverse	GATCCATTTAAATTCGAATTCTTAAGCGTAATCTGGAACATCGTATGGGTAGTCCCCCTTGGTGATGAGGTCCT
HA-MORC2 ΔC82	Forward	ACCTCCATAGAAGATCTAGAGCCGCCACCATGATGGCTTTCACAAATTACAGCAGT
	Reverse	GATCCATTTAAATTCGAATTCTTAAGCGTAATCTGGAACATCGTATGGGTACTTCAGAGGAAAAGATATTAGCTCA

Tables S2. Information of expression vectors used in this study

Plasmids	Sources	Vectors
Flag-MORC2	Self-cloned	pCDH-CMV-MCS-EF1-Puro
HA-MORC2	Self-cloned	pCDH-CMV-MCS-EF1-Puro/ pCDH-CMV-MCS-EF1-coGFP
HA-MORC2 ΔC82	Self-cloned	pCDH-CMV-MCS-EF1-Puro/ pCDH-CMV-MCS-EF1-coGFP
Lenti-CAS9	Addgene	LentiCas9-Blast
Lenti-guide	Addgene	LentiGuide-Puro

Table S3. sgRNAs targeting for MORC2 used in this study

Genes	Primers	Sequences
sgRNA #1	Sense	CACCGAGTAGACTCAGGTGTTCGCT
	Anti-sense	AAACAGCGAACACCTGAGTCTACTC
sgRNA #2	Sense	CACCGCCTCATGAAACGTGCGAGAC
	Anti-sense	AAACGTCTCGCACGTTTCATGAGGC

Tables S4. Information for primary antibodies used in this study

Antibodies	Vendors	Cat#	Species	WB	IHC	IP	IF
MORC2	Bethyl	A300-149	Rabbit monoclonal	√		√	
MORC2	Novus	NBP1-89295	Rabbit polyclonal		√		√
Flag	Sigma	F3165	Mouse monoclonal	√	√	√	√
HA	CST	3724S	Rabbit monoclonal	√	√	√	√
H2A	CST	12349	Rabbit monoclonal	√	√	√	√
H2B	CST	12364	Rabbit monoclonal	√			
H3	CST	4499	Rabbit monoclonal	√			
H4	Abcam	ab177840	Rabbit monoclonal	√	√	√	√
γH2AX	CST	9718	Rabbit monoclonal	√			
NPM1	Bethyl	A302-402A-1	Rabbit polyclonal	√		√	
hnRNPM1	Abcam	ab177957	Rabbit monoclonal	√	√		√
EGFR pY1068	CST	3777	Rabbit monoclonal	√			
HIF1α	Abcam	ab51608	Rabbit monoclonal	√	√	√	√
Actin	Sata Cruz Biotech	sc-47778	Mouse monoclonal	√	√	√	√
Vinculin	Sigma	V9131	Mouse monoclonal	√			
RAD51	Abcam	ab133534	Rabbit monoclonal	√	√	√	√
53BP1	Abcam	ab175933	Rabbit monoclonal	√	√		√
BRAC1	Abcam	sc-6954	Mouse monoclonal	√		√	√
